# Profound Hypomagnesemia Due to Proton Pump Inhibitor Use-Associated Wernicke’s Encephalopathy: A Case Report on Excitotoxicity

**DOI:** 10.7759/cureus.94687

**Published:** 2025-10-15

**Authors:** Benoit Delforge, Brieuc Volon, Yves Laurent, Charlotte Steinier

**Affiliations:** 1 Anesthesiology and Reanimation, Centre Hospitalier Universitaire (CHU) Université Catholique de Louvain (UCLouvain), Mont-Godinne, BEL; 2 Critical Care, Centre Hospitalier Universitaire (CHU) Hospital of Nivelles (HELORA), Nivelles, BEL; 3 Emergency Medicine, Centre Hospitalier Universitaire (CHU) Hospital of Nivelles (HELORA), Jolimont, BEL

**Keywords:** excitotoxicity, magnesium deficiency, neurologic manifestations, proton pump inhibitors, wernicke's encephalopathy, wernicke’s encephalopathy (we)

## Abstract

Hypomagnesemia is a frequently underdiagnosed condition. Its clinical consequences can be serious, with a wide variety of clinical presentations. Magnesium deficiency is often multifactorial, and this insufficiency can exacerbate or delay the recovery of other conditions. Magnesium is increasingly studied in the central nervous system for its regulation of excitotoxicity and its anti-apoptotic role. We describe the clinical case of a 50-year-old woman who presented to the emergency department with neurological symptoms characterized by dysarthria, headaches, photophobia, paresthesias, vertigo, multidirectional nystagmus, and gait instability. Her hospitalization revealed profound multifactorial hypomagnesemia (< 0.10 mmol/L). This condition results from diarrhea, chronic use of a proton pump inhibitor, alcohol consumption, and malnutrition. These electrolyte imbalances, along with the clinical presentation of Wernicke's encephalopathy, led to her admission to the intensive care unit. Her overall condition and neurological symptoms improved following the correction of her biochemical abnormalities, intravenous hydration, and thiamine supplementation. The patient was discharged from the hospital eight days later. In conclusion, hypomagnesemia is an electrolyte disorder whose neurological presentations remain rare but severe. Hypomagnesemia is a rare and insidious consequence of proton pump inhibitors and should be monitored frequently. The action of magnesium in the central nervous system is neuroprotective; it reduces inflammation and decreases excitotoxicity, a mechanism also involved in the pathophysiology of Wernicke's syndrome. This association is even more pertinent considering magnesium is necessary for the conversion of thiamine into its active form. Hypomagnesemia should be investigated more systematically and actively monitored, as the replenishment of the body's reserves can be time-consuming.

## Introduction

Since its discovery in 1920, magnesium has gradually been used at all levels of our hospital institutions as a cation acting against stress, cardiac dysrhythmias, and vasodilation. It has also been employed as an antihyperalgesic and as a neuroprotective agent in the context of strokes, preeclampsia, and the treatment of bronchoconstriction related to asthma and chronic obstructive pulmonary disease (COPD) [1,2]. This cation is the fourth most abundant found in the human body and plays a crucial role in numerous reactions, particularly in the homeostasis of certain other ions such as potassium (K) and calcium (Ca) [3]. This ion is predominantly intracellular, and its regulation occurs in three tissues: the kidneys, bones, and small intestine [4]. The magnesium level is subject to two main mechanisms: renal excretion and intestinal absorption. The etiology of hypomagnesemia can be multifactorial and is often complex. The normal serum concentration of magnesium typically ranges between 0.7 and 1.1 mmol/L. Magnesium imbalance may cause nonspecific symptoms, but in severe cases, it can lead to life-threatening complications, particularly cardiac and neurologic. A moderate "isolated" hypomagnesemia primarily induces nonspecific signs, including digestive disturbances (anorexia, nausea, etc.), fatigue, and increased stress [5]. The clinical manifestations of hypomagnesemia are frequently influenced by associated electrolyte disturbances and correlate with the severity and rapidity of the deficiency [3]. Neurological symptoms in hypomagnesemia result from a lowered neuronal activation threshold. They are often nonspecific but may progress to severe manifestations, including nystagmus, movement disorders, and neuromuscular hyperexcitability, such as Trousseau’s and Chvostek’s signs. Tetany or seizures may also occur, particularly when associated with hypocalcemia [3,6]. Magnesium exerts an inhibitory effect by antagonizing N-methyl-D-aspartate (NMDA) glutamatergic receptors and blocking calcium channels, thereby reducing the excitatory and secretory effects of calcium. Through these central mechanisms, magnesium is neuroprotective by decreasing excitotoxicity. This property of acting on excitotoxicity may be of interest in Wernicke’s encephalopathy, in which several studies demonstrate a mechanism of cell death through neuronal overactivation. Consequently, various articles in the literature highlight a link between magnesium levels and the prognosis for recovery from Wernicke's encephalopathy [7-9]. The prevalence of hypomagnesemia among patients taking proton pump inhibitors generally ranges between 14% and 27% (depending on the populations studied and the definitions applied), compared with about 13.5% in non-users [1]. Management relies on increased magnesium intake, administered intravenously (magnesium chloride or sulfate) or orally (~300 mg/day, 13 mmol/day) [10]. Treatment should be carried out long-term, as the replenishment of the body stores occurs slowly.

## Case presentation

The patient was a 50-year-old woman with multiple emergency department visits in recent months for abdominal pain, diarrhea, extremity paresthesias, nausea, vomiting, and asthenia. During prior admissions, imaging studies and laboratory evaluations were unremarkable, except for the finding of moderate hypomagnesemia. Several hours prior to her admission, she developed neurological symptoms, including dysarthria, incomprehensible speech, headaches, photophobia, vertigo, and gait instability. A fall with a likely period on the ground was highly suspected, and it could not be ruled out that the patient’s fall was related to a seizure episode.

Her past medical history included Hashimoto’s hypothyroidism treated with 100 µg L-thyroxine, untreated hypertension, and psoriatic arthritis managed with upadacitinib. Her metabolic syndrome was being managed with lipanthylnano and rosuvastatin. The patient smoked approximately 10 cigarettes per day and consumed 14 units of alcohol per week. It is noteworthy that she was also on a high dose of esomeprazole, 40 mg twice daily, with no clear indication justifying this dosage. Her treatment was further supplemented with duloxetine 30 mg daily and trazodone 100 mg in the evening.

Upon admission, she presented with tachycardia at 126 beats per minute, hypertension at 172/107 mmHg, a saturation of 100% on ambient air, and was afebrile. Her cardiovascular, pulmonary, and abdominal clinical examinations revealed no particular abnormalities. The neurological examination demonstrated tremors, agitation, a supple neck, equal and reactive pupils, multidirectional nystagmus, a sustained Barre test, and an unsustained Mingazzini test, with no sensory or motor disorders, and brisk reflexes. There was postural instability, but no dysmetria was observed during the finger-nose test. These neurological signs were likely attributable to the patient’s electrolyte imbalance.

Her blood tests upon admission revealed severe hypomagnesemia (< 0.10 mmol/L) associated with kidney failure, rhabdomyolysis, and hypokalemia (2.8 mmol/L). The results of the blood test are provided in the appendix (Table 1). Arterial blood gas analysis indicated respiratory alkalosis with a pH of 7.67, bicarbonate levels at 25 mmol/L, PCO₂ at 16 mmHg, and normal lactate levels. Thyroid tests showed good substitution balance.

**Table 1:** Patient's blood sample taken on admission U: units; L: liters; AST: aspartate aminotransferase; ALT: alanine aminotransferase; LDH: lactate dehydrogenase; CK: creatinine kinase; CRP: C-reactive protein; H: hours

Unit	Patient data	Normal range
CRP	1 mg/L	< 5 mg/L
Hemoglobin	14 g/dL	12.0-16.0 g/dL
Platelets (thrombocytes)	501 x 10^3^/µL	130-400 x 10^3 ^/µL
Leukocytes	29 x 10^3^/µL	4.5-11.0 x 10^3^ /µL
Neutrophiles	26 x 10^3^/µL (89 %)	45-75 % of white blood cells
Sodium	142 mmol/L	135-145 mmol/L
Potassium	2.8 mmol/L	3.4-5.0 mmol/L
Bicarbonates	18 mmol/L	18-23 mmol/L
Calcium, serum	2.65 mmol/L	2.1-2.6 mmol/L
Phosphorus	0.91 mmol/L	0.81-1.45 mmol/L
Magnesium	< 0.10 mmol/L	0.6-0.82 mmol/L
Creatinine (Cockcroft-Gault)	1.4 mg/dL (56 mL/min)	0.6-1.0 mg/dL
AST	183 U/L	9-25 U/L
ALT	66 U/L	7-30 U/L
LDH	562 U/L	< 270 U/L
CK	7734 U/L	40-150 U/L
Troponine T (high sensibility)	18 pg/mL	< 0.014 ng/mL
Troponine H+1	20.4 pg/mL	< 0.014 ng/mL

The electrocardiogram (ECG) revealed a regular sinus rhythm, normal atrioventricular conduction, narrow QRS complexes, and a downward deviation in the inferolateral region. Subsequently, a brain and abdominal CT scan with contrast injection was performed, revealing no particular abnormalities. A lumbar puncture was conducted and showed no red blood cells or white blood cells, with cultures proving negative, as did the multiplex PCR tests. The brain MRI was normal (3D sequences, axial T2, diffusion, susceptibility weighted imaging (SWI), and 3D T1 after contrast injection), and the electroencephalogram did not reveal any epileptiform activity.

The patient was admitted to the intensive care unit and treated with intravenous vitamin B1 and continuous magnesium sulfate over 24 hours. Following vitamin supplementation, the patient regained normal neurological functions, and electrolyte disturbances resolved. Upon discharge, renal function was normal, and rhabdomyolysis had resolved, with creatine kinase levels within the normal range. There was no documented indication for esomeprazole; thus, its dose was reduced to 20 mg per day. The patient was discharged from the hospital eight days later with the recommendation to continue oral magnesium supplementation.

## Discussion

Magnesium is essential for numerous enzymatic reactions, acting as both a cofactor and a substrate, and contributes to ionic balance [1,3]. Only ~1% of total body magnesium is extracellular (60% ionized, 33% protein-bound, 7% complexed), while most is stored in bone (60-70%) and muscle (25%) [1,5].

Because circulating magnesium represents only ~1% of body stores, serum levels provide a poor estimate of total status. The most reliable assessment is a 24-hour urinary magnesium excretion after an oral load; values <40% indicate depleted stores [4].

Magnesium balance is maintained through intestinal absorption and renal excretion. Intestinal uptake occurs via three pathways: (i) passive diffusion in the jejunum mediated by Claudin-16/19, (ii) active transport in the duodenum and ileocolic regions through TRPM6/7 channels, and (iii) active absorption of magnesium chelated to glycine, a recently described mechanism [3,11]. This process is influenced by luminal pH (enhanced in acidic conditions), body stores, PTH, and, to a lesser extent, vitamin D.

Renally, ~80% of magnesium is filtered and ~95% reabsorbed along the nephron, through both passive Claudin-mediated and active TRPM6-dependent mechanisms in the distal convoluted tubule [3]. The kidneys play the central role in homeostasis by upregulating claudin-16 and TRPM6 to increase reabsorption when needed, while chronic hypomagnesemia also triggers mobilization of bone reserves.

Hypomagnesemia can induce hypocalcemia due to functional hypoparathyroidism, as parathyroid hormone (PTH) secretion is magnesium-dependent [1]. Hypokalemia is observed in association with hypomagnesemia in 40% to 60% of cases, either due to concurrent potassium loss alongside magnesium loss (e.g., digestive losses) or from increased urinary potassium excretion in the distal convoluted tubule, induced by the inhibition of ROMK channels, a mechanism mediated by magnesium. This association can also result from dysfunction of the Na-K-ATPase pump, whose activity is influenced by intracellular magnesium levels [3,10,12,13].

The etiology of hypomagnesemia can be multifactorial and is often complex. A non-exhaustive list of etiologies is described in Table 2. However, it is important to differentiate between the different sources of hypomagnesemia. Renal etiologies include reduced reabsorption of filtered magnesium (diuretics, Gitelman's syndrome, Fanconi's syndrome, etc.), intestinal absorption disorders (celiac disease, bariatric surgeries), and medications, as well as various pathologies (hypothyroidism, endocrine disorders, alcoholism, etc.) [1,4,5,10]. A way to determine the origin of hypomagnesemia is by measuring the fractional excretion of magnesium (FEMg). Two origins are distinguished: renal (FEMg >2%) or extrarenal (FEMg <2%). Moreover, hereditary forms, generally associated with renal tubulopathies, are differentiated from acquired forms, which can be of metabolic, toxic, or drug-related origin [1]. 

**Table 2:** A non-exhaustive list of hypomagnesemia etiologies References: [1,3-5]

Renal etiologies	Extrarenal etiologies
Fanconi’s syndrome	Intestinal malabsorption
Gitelman’s syndrome, Bartter's syndrome, Familial hypomagnesemia	Alcoholism
Hypercalciuric hypomagnesemia	Diarrhea or vomiting
Mitochondrial hypomagnesemia	Malnutrition
Diuretics (loop diuretics and some thiazide diuretics)	Hungry bone syndrome
Hypercalcemia, Hyperparathyroidism	Intestinal neoplasia
Osmitic diuresis	Total parenteral nutrition
Polyuria/acute tubular necrosis/post-obstructive uropathy	Bariatric surgery or previous bowel resection
Drug etiologies	Prolonged exercise
Proton pump inhibitors (PPIs)	Proteinuria
Estimated glomerular filtration rate (eGFR) inhibitors	Stress, sepsis, chronic illness, and inflammatory bowel disease
Antibiotics (aminoglycosides, amphotericin B)	Acute pancreatitis
Massive blood transfusions	Milk-alkali syndrome
Platinum derivatives (e.g., cisplatin)	Syndrome of inappropriate antidiuretic hormone secretion (SIADH), hypothyroidism, primary and secondary hyperaldosteronism
Pentamidine	Hyperphosphatemia and renutrition syndrome
Cortison	Hypovitaminosis D
Calcineurin inhibitors: cyclosporine and tacrolimus	Acidosis (increased ionized magnesium fraction)

In our patient, the use of proton pump inhibitors likely played a significant role in her profound hypomagnesemia. Proton pump inhibitors are widely prescribed medications, and their safety and efficacy are well established. However, this treatment is not without side effects, one of the lesser-known being hypomagnesemia accompanied by functional hypoparathyroidism and hypocalcemia [1,14,15]. The incidence of this side effect is probably underestimated, given the relatively asymptomatic nature of magnesium imbalance [16]. The mechanism leading to this hypomagnesemia is not fully understood but likely involves reduced intestinal absorption of magnesium through inhibition of TRPM6 and TRPM7 channels, as well as inhibition of the H+/K+ ATPase pump in the colon, resulting in altered intestinal luminal pH and decreased active transport of magnesium via TRPM6 [3,17]. This mechanism is probably not solely intestinal, as this side effect is not observed in renal transplant patients [18]. Generally, treatment consists of oral supplementation and reduction of the proton-pump inhibitor dose or switching to H2 blockers. The effect of proton pump inhibitors on magnesium levels is rapidly reversible, typically within about four days [1].

Magnesium exerts neuroprotective effects by antagonizing NMDA and contributes to neurotransmission, neuromuscular conduction, and blood-brain barrier integrity, while also modulating inflammation through NF-κB, SOD, and substance P [19,20]. By regulating neuroinflammation, magnesium has been implicated in neurodegenerative diseases (Parkinson’s and Alzheimer’s) and studied as a preventive or therapeutic agent in depression, anxiety, migraine, and chronic pain [20-23]. In epilepsy, where excessive glutamatergic activity plays a central role, magnesium may have a modulatory effect; however, its role as an antiepileptic remains debated and requires further randomized studies [24].

Theoretically, hypomagnesemia can enhance glutamatergic activity, leading to excitotoxicity, oxidative stress, and ultimately cell death [6,20,25]. Thus, in the case of stroke, some studies demonstrate a protective role of magnesium against excitotoxicity [22,26]. Excitotoxicity is defined as the pathological process by which nerve cells are damaged and killed due to excessive stimulation by excitatory neurotransmitters such as glutamate. This phenomenon primarily involves the overactivation of glutamate receptors, particularly N-methyl-D-aspartate receptors (NMDARs), leading to a cascade of neurotoxic events. These events include sustained calcium influx, oxidative stress, mitochondrial dysfunction, and activation of pro-apoptotic pathways, ultimately resulting in neuronal injury and cell death [27].

Wernicke's encephalopathy is caused by a deficiency of vitamin B1 (thiamine), which can lead to various symptoms such as the triad described by Carl Wernicke: confusion, oculomotor disturbances (ophthalmoplegia and nystagmus), and ataxia. Other symptoms may also be present, such as motor deficits, a glove-and-stocking sensory loss, and peripheral ataxia [28,29]. Diagnosis can be made through thiamine measurement or visualization of certain signs on MRI.

In Wernicke's encephalopathy, several studies demonstrate a mechanism of cell death through excitotoxicity; thiamine deficiency causes alterations in glutamate uptake via changes in the function of astrocytic transporters EAAT1 and EAAT2, leading to excitotoxicity [30]. This mechanism is particularly well established in rats, and the administration of NMDA receptor inhibitors reduces this excitotoxicity [31].

Several sources present magnesium as an important agent in the resolution of Wernicke's encephalopathy by acting as a cofactor for the conversion of thiamine into active metabolites [32,33]. Moreover, its antagonistic action on NMDA receptors and its anti-excitotoxicity properties enhance its ideal profile for treating the condition. Several cases of Wernicke's encephalopathy have been reported as refractory despite thiamine supplementation until magnesium levels were corrected [7]. A publication has demonstrated a delay in correcting serum thiamine levels and symptom resolution in several patients with hypomagnesemia compared to patients with normal magnesium levels [8]. Another case involved a patient with Wernicke's encephalopathy who did not respond to thiamine supplementation; the clinical condition improved after correcting his hypomagnesemia [9].

## Conclusions

The patient presented with Wernicke’s encephalopathy in the context of profound hypomagnesemia, likely multifactorial in origin, with chronic double-dose proton pump inhibitor therapy contributing significantly. Although cases of Wernicke’s encephalopathy associated with hypomagnesemia have been reported, the shared mechanism of excitotoxicity has, to our knowledge, not been explicitly emphasized. This association is particularly relevant given that magnesium is required for the conversion of thiamine into its active form, and hypomagnesemia may therefore contribute to thiamine deficiency. Our case suggests a possible, though still hypothetical, connection between excitotoxicity, hypomagnesemia, and Wernicke’s encephalopathy, a link that has been proposed by several studies but not yet firmly established. Monitoring magnesium levels should be performed in cases of Wernicke's encephalopathy, knowing that hypomagnesemia worsens the rate of correction and increases excitotoxicity, and that magnesium is crucial for correcting thiamine deficiency.
